# Increasing the biomolecular relevance of cell culture practice

**DOI:** 10.1186/s12929-024-01095-6

**Published:** 2025-01-03

**Authors:** Raluca E. Ghebosu, Lawrence Hui, Joy Wolfram

**Affiliations:** 1 Australian Institute for Bioengineering and Nanotechnology, The University of Queensland, Brisbane, 4072 Australia; 2 School of Chemical Engineering, The University of Queensland, Brisbane, QLD 4072 Australia

**Keywords:** Chemically defined medium, Fetal bovine serum (FBS), Plasma, Serum, Supplement

## Abstract

The biomolecular relevance of medium supplements is a key challenge affecting cell culture practice. The biomolecular composition of commonly used supplements differs from that of a physiological environment, affecting the validity of conclusions drawn from in vitro studies. This article discusses the advantages and disadvantages of common supplements, including context-dependent considerations for supplement selection to improve biomolecular relevance, especially in nanomedicine and extracellular vesicle research.

## Background

Cell culture studies have been a cornerstone of scientific research since the first isolation of nerve cells from a frog in 1907 [1]. Conventional cell culture practice often entails two-dimensional (2D) plastic dishes filled with medium containing bovine-based supplements [2]. The 2D cell culture format fails to accurately depict interactions between cells and their extracellular surroundings, impacting other processes such as cell signaling and division [3, 4]. In addition, physiologically relevant fluid dynamics are not implemented in conventional cell culture practices, overlooking various effects on cells, such as shear stress [3]. Moreover, bovine-based supplements fail to accurately depict a physiologically relevant species-specific environment. Taken together, the limitations of conventional cell culture practices span physical and molecular factors. This article focuses on the biomolecular relevance of cell culture practice by comparing the composition and impact of common supplements. The possible implications of physiologically irrelevant supplements are discussed, especially in the context of nanomedicine and extracellular vesicle research studies, which are susceptible to artificial results due to use of inappropriate cell culture media.

Fetal bovine serum (FBS) is a widely used cell culture supplement obtained from fetuses during the slaughter of pregnant cows [5]. The rich nutrient and growth factor content of FBS is used for the maintenance and proliferation of a wide range of human and animal cell lines [5–7]. Another widely used cell culture supplement is chemically defined media, which contain a distinct mixture of biomolecules, such as growth factors, lipids, and amino acids, required for cell growth [8–11]. Human serum has also been used as a cell culture supplement, displaying higher compatibility with clinical-grade cell manufacturing than FBS and improved biomolecular relevance for human cells compared to FBS or chemically defined media [12–15]. Human serum is derived from the supernatant of coagulated blood, consequently having a biomolecular profile representative of a pathophysiological environment (blood clotting). Human plasma is an overlooked cell culture supplement that is closely representative of the physiological extracellular environment. This article focuses specifically on the disadvantages and advantages of FBS, chemically defined media, human serum, and human plasma, noting that several other cell culture supplements have also been developed and reviewed elsewhere.

### Biomolecular relevance

**Fig. 1:**
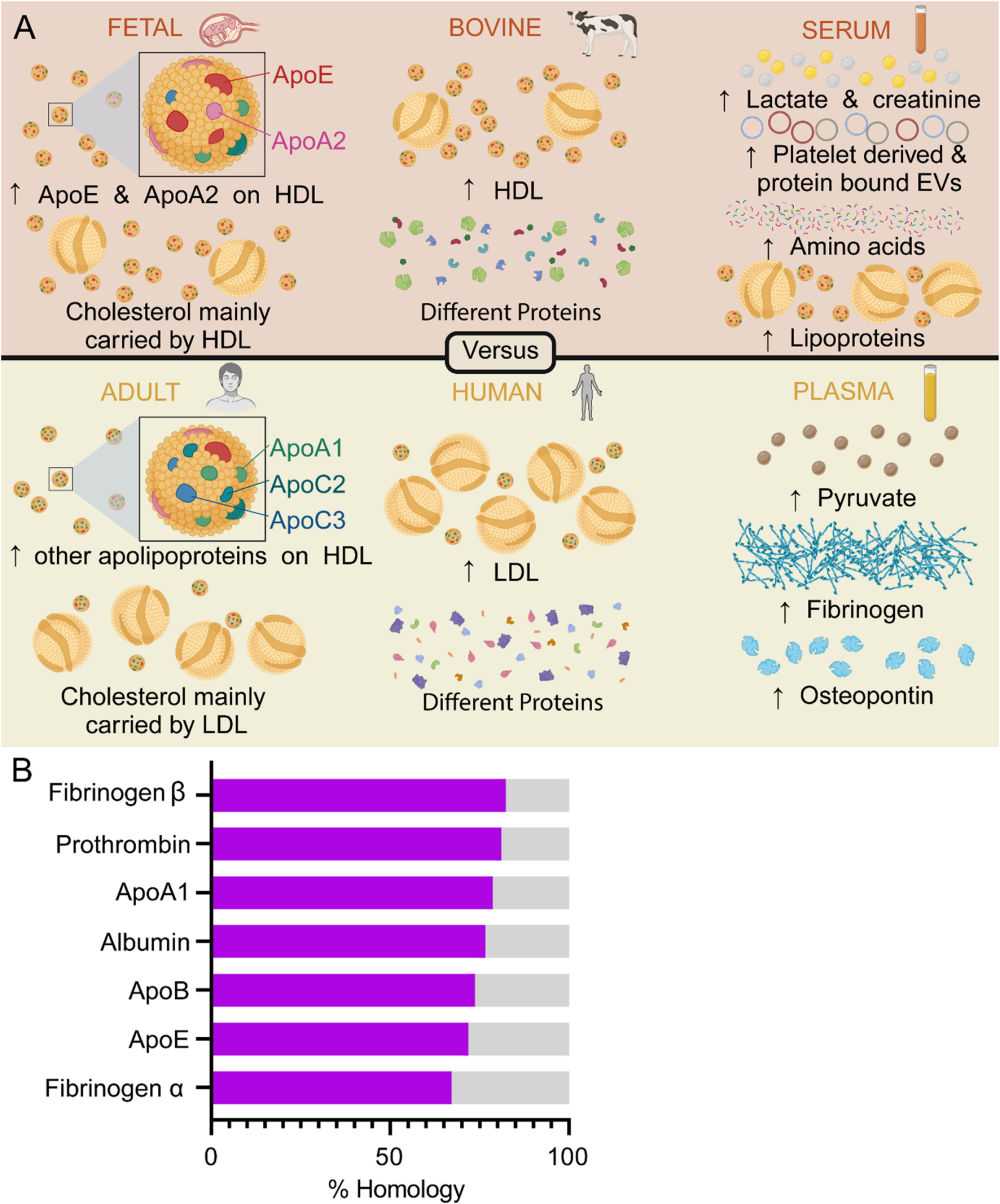
Biomolecular differences between various cell culture supplements. **A** Comparison between fetal bovine serum and adult human plasma in the three major areas of differences (fetal versus adult; bovine versus human; serum versus plasma). Arrows indicate an increase (↑) or decrease (↓) in the levels of biomolecules based on developmental stage (fetal versus adult), species (bovine versus human), and non-coagulated/coagulated state (serum versus plasma). **B** Approximate percentage of homology between abundant bovine and human plasma proteins, determined by UniProt. [48] *Apo*, apolipoprotein; *EV* extracellular vesicle, *HDL*, high-density lipoprotein, *LDL* low-density lipoprotein

FBS contains a variety of biomolecules and the complex combination of factors responsible for promoting cell maintenance and proliferation have yet to be fully determined [8, 16]. It is important to consider the biomolecular relevance of FBS as a supplement for non-bovine cell lines. FBS is poorly reflective of adult human biomolecules due to fetal, bovine, and serum origin. Therefore, the extensive use of FBS in cell culture calls into question the applicability and validity of in vitro studies [16, 17]. FBS reflects a biomolecular environment corresponding to fetal physiology, which is distinct to that of an infant or adult (Fig. 1A). The biomolecular composition of fetus blood differs substantially from that of an adult. For example, fetus plasma has differing proteomic and lipidomic profiles compared to adult plasma [18–20]. Specifically, high density lipoprotein (HDL) is the major carrier of cholesterol in fetus cord blood, compared to low density lipoprotein (LDL) in maternal serum [19]. Furthermore, apolipoprotein (Apo)E and ApoA-II are enriched on fetal HDL [19], while the levels of many lipids are substantially lower than in maternal plasma (Fig. 1A) [18, 19]. Fetal cord blood also contains substantially higher levels of biomolecules, such as albumin, hemoglobin, platelet factor 4, and vitamin D-binding protein (Fig. 1A) [19]. Therefore, while there are several embryonic/fetal cell lines in use, culturing other cell lines with fetal supplements, such as FBS, is unlikely to reflect a physiologically relevant biomolecular environment. A comparison of various head and neck carcinoma cells grown in FBS, newborn calf serum (< 14 days old), and calf serum (< 12 months old), revealed substantial differences in cell proliferation based on the used sera [21]. Cells grown with newborn calf serum showed delayed attachment (36 h), altered morphology, and poor proliferation compared to FBS (~ 80% lower at 72 h) [21]. Similarly, calf serum led to lower short-term and long-term proliferation than FBS (~ 50% lower) and morphological changes in most of the assessed cell lines; although the supplement outperformed newborn calf serum [21]. These results further emphasize the importance of considering the effects of the supplement-associated development stage on cell culture.

In addition to reflecting the biomolecular environment of fetal physiology, FBS is representative of bovine physiology. Human and bovine serum contain many of the same proteins, but they differ in homology (Fig. 1B), which could impact various biological processes, including those relying on precise receptor-ligand interactions. The functional implications of species-specific biomolecules in cell culture studies remain poorly understood and are likely to be assay-dependent. In addition to homology issues, the overall biomolecular composition and abundance of plasma/serum varies between species. For example, bovine serum has differing lipid and amino acid profiles compared to human serum (Fig. 1A) [22–24]. Human serum-derived lipoproteins contain different compositions of triglycerides, cholesterol, cholesterol esters, and phospholipids compared to their bovine counterparts [22, 24].

The limitations of FBS in terms of developmental stage and species-specific characteristics are further exacerbated by the use of serum, which reflects the biomolecular composition of a specific pathophysiological environment (for example, wound healing). The generation of serum alters the biomolecular composition of blood due to coagulation. For example, human serum contains higher levels of lactate, creatinine, lipoproteins, vitamin D binding proteins, and amino acids compared to plasma (Fig. 1A) [25–31]. Serum fails to accurately represent the biomolecular composition of the native extracellular space, as most cells are surrounded by interstitial fluid, which more closely resembles plasma than serum.

The differing proteomic and metabolomic profiles of serum and plasma can be attributed to the presence of anticoagulants in plasma and distinct processing procedures [25, 26, 29, 30, 32, 33]. Addition of anticoagulants, such as ethylenediaminetetraacetic acid (EDTA), citrate, and heparin, to plasma may also partially inhibit proteolytic activity, favoring biomolecular stability due to reduced protein and peptide degredation [34]. The biomolecular composition also differs depending on the type of anticoagulant, especially in terms of levels and composition of fibrinogen, complement C3 and C4, and bradykinis [34]. Although plasma is closely representative of normal physiology, a disadvantage of plasma is susceptiblity to clotting, especially in media containing high levels of calcium. Potential clotting of plasma can be overcome through the addition of heparin, which is already used in some media formulations [35]. It is important to note that human plasma reflects the extracellular environment of endothelial and hematopoietic cells. Other cell types, which are not in direct contact with the blood, are exposed to an interstitial fluid, that possesses a tissue-dependent biomolecular composition. The interstitial fluid usually contains lower concentrations of circulating biomolecules than plasma, as macromolecules, such as albumin and lipoproteins, are required to undergo endothelial transcytosis to enter the interstitial space in tissues with continuous vasculature [36].

Finally, chemically defined media contains a mixture of biomolecules that promote cell growth, however, the exact composition of these formulations often remains unknown to users due to commercial proprietary reasons [16]. Compared to plasma and serum-based supplements chemically defined media contain minimal biomolecular diversity, failing to accurately mimic a physiological environment. Additionally, chemically defined media can vary substantially in composition, leading to different gene expression in cultured cells [37].

Another key consideration in terms of biomolecular composition of cell culture supplements is heterogeneity. Extensive batch-to-batch variability has been documented, which affects consistent cell growth and leads to reproducibility challenges in research [16, 17]. Inter-supplier variability is another challenge, with FBS displaying differing metabolomic profiles and cellular effects depending on the commercial vendor [17]. For example, FBS-induced secretion of inflammatory cytokines in epithelial cells varies depending on the brand [17]. For human blood products, age and blood group variability can often be accounted for, but unknown genetic and lifestyle factors impact the biomolecular composition, leading to extensive heterogeneity [38–40]. Pooling of human serum or plasma is one approach to reduce variability [15, 41, 42]. An advantage of chemically defined media is decreased batch-to-batch variability [10, 11] compared to FBS, human serum, and plasma.

Selection of supplements for clinical manufacturing of cell therapy poses additional challenges. In general, FBS is deemed unsuitable for clinical cell manufacturing [13–15] due to various reasons, including potential transmission of zoonic bacteria/viruses and prion diseases [43–45]. Pathogenic transmission is possible with human serum and plasma, and these supplements are often screened for select viral diseases, such as human immunodeficiency virus and hepatitis B virus [25, 44]. Residual FBS may also induce xenogeneic responses [43, 45, 46], while residual human serum and plasma could induce allogeneic immune responses [47]. Allogeneic reactions caused by residual human serum and plasma could be mitigated by using universal donors (AB) [14].

### Consequences of biomolecular relevance

The use of supplements with non-physiologically relevant biomolecular compositions substantially impacts cell culture studies. For example, serum lacks fibrinogen [25], which is a major component in the coagulation cascade and has also been demonstrated to impact cell growth, motility, and intra/extravasation. Interactions with fibrinogen protected cancer cells from natural killer cell-mediated cytotoxicity [49], a phenomenon that could have gone undiscovered with cell culture supplements, such as FBS and serum. Furthermore, fibrinogen signaling can also activate pro-inflammatory pathways, regulate innate immune responses, and impact a variety of autoimmune and neurological diseases [50, 51]. Specifically, fibrinogen has also been shown to promote cell migration of smooth muscle cells [52], promote inflammation in peripheral blood mononuclear cells [53] and alter microvascular permeability [54]. Therefore, the absence of fibrinogen in cell culture studies may lead to results and conclusions that are poorly reflective of an in vivo environment. Osteopontin is another factor that is substantially depleted in human serum compared to plasma [25]. Osteopontin is an extracellular matrix protein, first identified in bone tissues [55] and later shown to regulate immune cell function, angiogenesis, and cancer progression [55, 56]. Taken together, low levels of certain biomolecules, such as osteopontin and fibrinogen, in the most widely used cell culture supplements may lead to false interpretations of cell function and an inability to assess physiologically relevant phenomena.

**Fig. 2:**
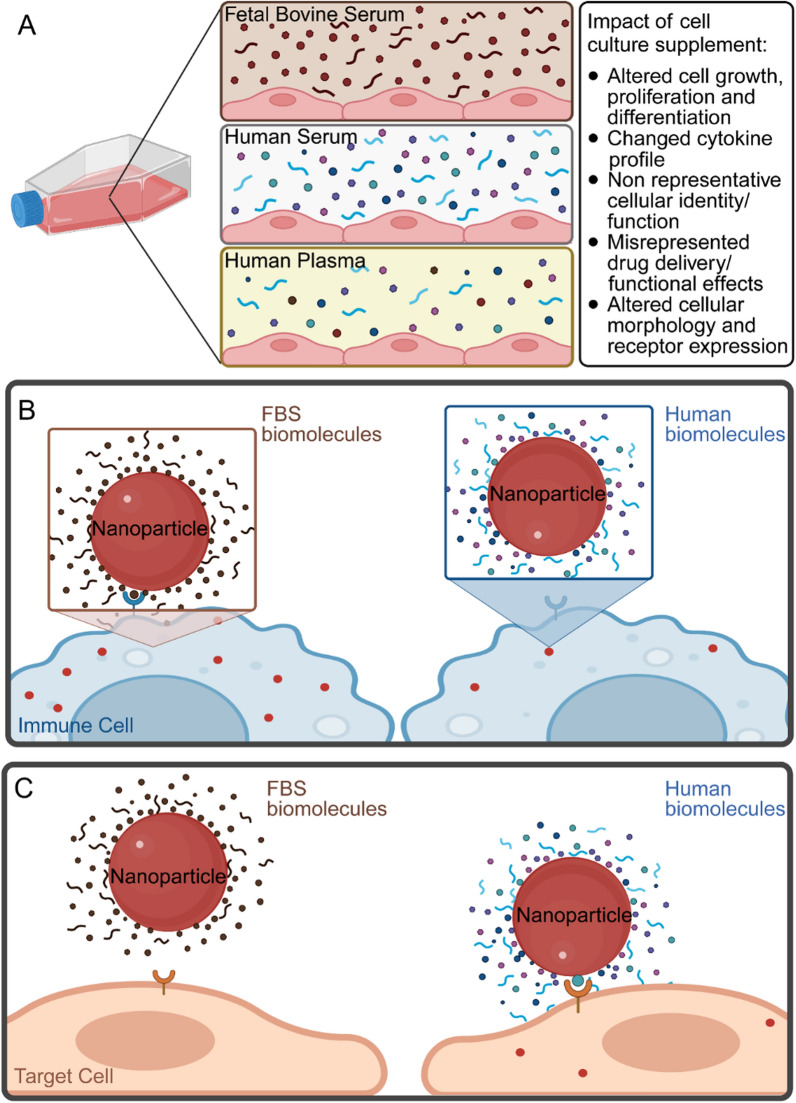
Altered biomolecular composition of cell culture supplements can impact immune clearance and target cell uptake. **A** Potential disadvantageous (non-physiologically relevant) effects of cell culture supplements. Cell culture supplements impact biomolecular identity of drug delivery systems such, as nanoparticles, by affecting immune clearance (**B**) and target cell uptake (**C**)

In addition to reduced levels of specific proteins, certain biomolecules are enriched in serum compared to plasma, including metabolites and lipids, which are vital for cell growth, but also affect cell function and fate [57–60]. Supplementation of cell culture medium with exogenous lipids impacts proliferation, viability, antibody secretion, and the membrane composition of cells [37, 60, 61]. Serum free alternatives have been employed to study endogenous lipid synthesis in cell culture [60]. Melanoma cells cultured in a chemically defined medium without lipids displayed substantially increased expression of de novo fatty acid, cholesterol synthesis genes, and the LDL receptor compared to supplementation with FBS [60]. However, conditions without exogenous lipids are unlikely to reflect a physiological environment. Taken together, both an excess and deficiency of biomolecules compared to a physiological environment is likely to alter cell function (Fig. 2A).

Initial drug discovery studies are often performed in cell culture studies. Interactions between drugs and the surrounding biomolecular environment impacts clearance, biodistribution, safety, and efficacy. Various therapeutic agents were shown to bind to biomolecules in the blood, such as albumin, lipoproteins, and glycoproteins [59]. Nanomedicines are especially susceptible to interactions with biomolecules in the blood and extracellular space, as they have large surface areas [62]. The field of nanomedicine has rapidly expanded over the past few decades with several nanomedicines approved for clinical use [63, 64], making it especially important to emphasize best practices for studies with nanoparticles [65]. Nanoparticles exposed to either human plasma, mouse plasma, or FBS display differing interactions with biomolecules [66–69]. Such interaction change the physical and biological identity of nanoparticles, including size, surface charge, cellular uptake, and immune clearance [66, 68, 69] (Fig. 2B-C). For example, synthetic lipid nanoparticles exposed to FBS showed higher uptake in human epithelial cervical cancer cells than those exposed to human serum [69]. Therefore, it is especially important to consider the impact of supplement selection (for example, species matching) on nanoparticle effects in cell culture.

In addition to nanomedicine, the field of extracellular vesicles is highly susceptible to artificial cell culture results due to inappropriate supplement selection and a lack of standardization within the field [70, 71]. Extracellular vesicles are biological nanoparticles, with one or several lipid bilayers, released by all cells [72]. Over the past decade, the field of extracellular vesicles has grown exponentially and several extracellular vesicle products are in clinical trials [73, 74]. Similar to synthetic nanoparticles, extracellular vesicles have a large surface area, which promotes interaction with surrounding biomolecules, especially lipoproteins that are present in blood-derived supplements [75, 76]. A study demonstrated that cancer cell-derived extracellular vesicles induce TNF-α expression in monocytes cultured in supplements depleted of lipoproteins [70]. However, the lack of lipoproteins is physiologically irrelevant, as lipoproteins are abundant in blood and present in the interstitium of tumors and healthy tissues [70]. Supplementing the culture medium with physiological levels of the lipoprotein, LDL, led to the opposite findings, where the extracellular vesicles reduced TNF-α expression in monocytes [70]. Therefore, artificial results and conclusions can arise when the extracellular space lacks physiologically relevant biomolecular components. The cellular uptake of various cancer extracellular vesicles was also altered in a cell type-dependent manner in the presence of LDL [70]. Specifically, cancercell-derived extracellular vesicles displayed lower uptake in human brain microvascular endothelial cells but higher uptake in monocytes following the addition of LDL [70]. Adsorbed biomolecules on the extracellular vesicle surface are also likely to impact immunological recognition and clearance [77]. Taken together, a lack of physiologically relevant biomolecules fails to accurately portray extracellular vesicle and nanoparticle cell culture interactions in vivo. In general, the use of human plasma as a cell culture supplement more accurately depicts nanoparticle and extracellular vesicle interactions with a physiologically relevant environment.

The selection of cell culture supplements is also a key consideration in the isolation of extracellular vesicles from conditioned media. Serum and plasma contain levels of extracellular vesicles that far outnumber those produced by cells in culture [70]. Additionally, these supplements also contain large amounts of contaminants, such as lipoproteins, protein aggregates, and polysaccharides, that are challenging to separate from extracellular vesicles [78, 79]. Therefore, supplements should ideally be depleted from extracellular vesicles and other similar sized contaminants to enable isolation and study of cell culture-produced extracellular vesicles. However, various cell types, such as glioblastoma, neuroblastoma, cervical carcinoma, human embryonic kidney cells, and myoblasts demonstrated decreased proliferation when extracellular vesicles were depleted from FBS and human serum [80, 81]. Additionally, the depletion of extracellular vesicles from FBS altered the differentiation of myoblasts [80]. Such altered cell characteristics is likely to affect the composition and function of cell culture-specific extracellular vesicles, which should be taken into consideration [70].

In addition to extracellular vesicle-depleted blood-derived supplements, chemically defined media have also been used for cell culture-derived extracellular vesicle production. One study showed that a chemically defined low-protein medium (Opti-MEM) reduced cell proliferation compared to extracellular vesicle-depleted FBS, but resulted in increased extracellular vesicle production, which is common during cell stress [82]. Notably, the extracellular vesicles produced with Opti-MEM differed in protein composition, in particular displaying increased levels of stress-related proteins, compared to those produced in extracellular vesicle-depleted FBS [82]. New types of chemically defined media have been developed to improve cell viability, while ensuring high levels of extracellular vesicle production [83]. It is important to note that chemically defined media can also be a source of contaminants in extracellular vesicle isolation studies. For example, subjecting a chemically defined medium to ultracentrifugation, a common extracellular vesicle isolation technique, led to the formation of protein aggregates that co-pelleted with extracellular vesicles, contaminating the samples [84].

In addition to using extracellular vesicle-depleted supplements and chemically defined media, many studies starve cells (lack of exogenous proteins/lipids) during the period of extracellular vesicle generation [85, 86]. Starvation will result in more drastic stress-induced effects on cells compared to supplements that contain proteins and/or lipids. Consequently, starvation-induced cell stress, including apoptosis, can substantially increase extracellular vesicle production and change extracellular vesicle characteristics [82], which may fail to reflect a physiologically relevant environment. Taken together the effects on supplement selection should be carefully considered in the extracellular vesicle field to avoid the production of contaminated or physiologically irrelevant samples that result in false/inaccurate findings about biological effects.

### Considerations other than biomolecular relevance

Non-physiologically representative cell culture supplements have many disadvantages, including abnormal cell proliferation, differentiation, morphology, signaling, and responses to exogenous agents, such as drugs. However, biomolecular relevance should not be considered in isolation from other factors, such as ethics, logistics, and protocol standardization, when selecting the most suitable cell culture supplements. In fact, the use of human-derived products as cell culture supplements poses unique logistical and ethical challenges. While human serum and plasma can be derived from pre-existing infrastructure, such as blood banks, their usage in cell culture competes with patient-driven demand for blood products. However, sourcing blood products that have been deemed clinically unsuitable (for example, lengthy storage times) has been recommended as a potential solution to avoid competing with patient demand [87]. It is also important to note that the use of human blood products requires adherence to informed consent, reporting, traceability and confidentiality protocols [87]. The use of FBS also poses logistical and ethical concerns. For example, suppliers are tasked with sustainably meeting the global demand for FBS, which requires two million bovine fetuses each year [7]. There is also a lack of regulatory control in the production of FBS [5, 16], which raises ethical issues concerning the treatment of cows during the collection process [8]. Chemically defined media have less ethical concerns compared to FBS, human serum, and plasma [10, 11].

In addition to logistical and ethical considerations of cell culture supplements, other advantages and disadvantages exist, including those related to protocol standardization and cell culture maintenance. For example, many cell culture protocols have been standardized based on FBS to ensure consistent results, including diagnostic assays [88–90]. Additionally, FBS has proven suitability for culturing a broad range of cell types. In some cases, human serum displays an improved ability to sustain cell proliferation and/or differentiation compared to FBS [44, 47, 91, 92]. For example, the use of human serum decreased the doubling times of adipose mesenchymal stromal cells [44] and chondrocytes [47] by approximately 20 h and up to ten days, respectively. For other cells, such as cervical cancer cells, human serum resulted in similar metabolic activity, proliferation, and cell migration as FBS, but enhanced spheroid formation and caused a 23–43% increase in cell invasion [91]. Select studies have shown that human plasma is comparable to FBS and human serum in terms of cell proliferation and/or differentiation [13, 46, 93, 94]. In the case of adipose mesenchymal stromal cells, proliferation and endothelial differentiation was similar in human plasma and FBS [93]. Dendritic cells also displayed similar cell viability when grown in human plasma compared to human serum [13]. However, periodontal ligament cells grown in plasma displayed substantially higher cell proliferation compared to conditions with FBS [94]. On the contrary, comparative studies showed that chemically defined media are unable to sustain cell growth/proliferation as effectively as FBS and human serum [9, 95]. The overall strengths and limitations of the discussed cell culture supplements are summarized in Table 1 [25].

**Table 1:** Summary of key challenges and limitations of FBS, human serum, human plasma and chemically defined media

Supplement	Fetal bovine serum	Human serum	Human plasma	Chemically defined
LOGISTICAL				
Supply	Limited cattle inventory	Limited clinical inventory	Limited clinical inventory	Available
Accessibility	Higher	Lower	Lower	Higher
Commercial cost	Low	High	High	Low
BIOLOGICAL RELEVANCE				
Human physiology	Not representative	Representative	Representative	Not representative
Post-fetal physiology	Not representative	Representative	Representative	Not representative
Cell physiology	Not representative	Not representative	Representative	Not representative
Compatibility with cell therapy	Low	High	High	High
Immunogenic responses	Possible xenogeneic immune responses	Possible allogeneic immune responses	Possible allogeneic immune responses	Not applicable
CELL EXPANSION				
Cell proliferation	Higher	Higher	Lower	Lower
Complement lysis	Unlikely (fetal origin)	Possible	Possible	Not applicable
Clotting	Not applicable	Not applicable	Possible	Not applicable
Viral (human) contaminants	Unlikely	Possible	Possible	Unlikely
Batch variability	High	High	High	Low
ETHICAL				
Human and animal ethics	Animal rights (for example, lack of anesthesia)	Competing with patient needs	Competing with patient needs	Not applicable

In most cases, improved physiological relevance can be achieved by matching the supplement to the species, development stage, and health/disease state of the cells that are being studied. However, this is highly context-dependent, and in some cases, the modification of supplements through depletion of certain components may be necessary to obtain ideal results, such as in extracellular vesicle isolation studies. The use of machine learning software and artificial intelligence has become more prominent for the optimization of cell culture media for cell maintenance and meat cultivation [96–98]. These technologies are promising for the development of media formulations with enhanced experimental applicability and biomolecular relevance.

## Concluding remarks

There are a variety of cell culture supplements available, each with distinct advantages and disadvantages, spanning biomolecular relevance, logistics, ethics, and protocol standardization. The differing biomolecular composition of FBS, human serum, plasma, and chemically defined media leads to varying results in cell culture studies. The heterogeneity of blood products can also impact the reproducibility of cell culture studies. The precise content of chemically defined media can be controlled but is scarcely representative of a physiological environment with diverse biomolecular components. The failure of conventional cell culture to represent physiological conditions is a key limitation that impacts the validity of conclusions, especially in fields, such as nanomedicine and extracellular vesicle research. Therefore, it is critical to assess the importance of extracellular biomolecules in cell culture studies and make efforts to use the most physiologically relevant supplements. Increasing the biomolecular relevance of cell culture practice through machine learning software and artificial intelligence will pave the way for future discoveries and uncover previously unknown biological mechanisms.

## Data Availability

The datasets generated during and/or analyzed during the current study are available in the UniProt repository, UniProt.

## Abbreviations

2D: Two dimensional
ApoA-1: Apolipoprotein A-1
ApoE: Apolipoprotein E
EDTA: Ethylenediaminetetraacetic acid
FBS: Fetal bovine serum
HDL: High-density lipoprotein
LDL: Low-density lipoprotein
NMDA: N-methyl-D-aspartate
TNF-α: Tumor necrosis factor‐α
